# Prevalence of hypobetalipoproteinemia and related psychiatric characteristics in a psychiatric population: results from the retrospective *HYPOPSY* Study

**DOI:** 10.1186/s12944-018-0892-4

**Published:** 2018-11-06

**Authors:** Bertrand Cariou, Gaëlle Challet-Bouju, Céline Bernard, Marie Marrec, Jean-Benoit Hardouin, Charlotte Authier, Kalyane Bach-Ngohou, Christophe Leux, Matthieu Pichelin, Marie Grall-Bronnec

**Affiliations:** 1 CHU Nantes, l’institut du thorax, INSERM, CNRS, UNIV Nantes, CHU Nantes, F-44000 Nantes, France; 2 CHU de Nantes, CIC Endocrino-Nutrition INSERM UMR 1413, l’nstitut du thorax, F-44000 Nantes, France; 3 CHU Nantes, Service d’Addictologie et de Psychiatrie, F-44000 Nantes, France; 4 Université de Nantes, Université de Tours, Inserm UMR 1246-SPHERE, F-44000 Nantes, France; 5 CHU Nantes, DRCi, Plateforme de Méthodologie et de Biostatistique, F-44000 Nantes, France; 6 Centre d’examens de santé de la Caisse Primaire d’Assurance Maladie de Loire-Atlantique, St Nazaire, F-44600 Saint-Nazaire, France; 7 CHU Nantes, Service de Biochimie, F-44000 Nantes, France; 8 INSERM1235, Université de Nantes, Institut des Maladies de l’Appareil Digestif, F-44000 Nantes, France; 9 CHU Nantes, Service d’Information Médicale, F-44000 Nantes, France; 10 Clinique d’Endocrinologie, Maladies Métaboliques et Nutrition, Hôpital Guillaume & René Laennec, Boulevard Jacques Monod, Saint-Herblain, 44093 Nantes Cedex 1, France; 11 Present address: CHU Sud Reunion, Site Saint-Pierre, Avenue président F Mitterrand, BP 350, 97448 ST Pierre Cedex, La Reunion France

**Keywords:** Hypobetalipoproteinemia, LDL cholesterol, Autism spectrum disorders, Hetero-aggression, Schizophrenia

## Abstract

**Background:**

Hypobetalipoproteinemia (HBL) is defined by plasma concentrations of LDL-cholesterol (LDL-C) lower than the fifth percentile for age and sex. Several psychiatric symptoms have been reported in association with HBL. The objective was to assess the prevalence of primary HBL in patients hospitalized in a psychiatric population and to better characterize the related psychiatric disorders.

**Methods:**

HYPOPSY is a retrospective study including 839 adults hospitalized in the Psychiatry department of Nantes University Hospital during the year 2014, except patients with eating disorders. The prevalence of primary HBL was defined by a plasma LDL-C concentration ≤ 50 mg/dL. Secondary causes of HBL were excluded after a review of medical records (*n*=2). Related-psychiatric disorders in patients with and without HBL were recorded using the ICD-10 classification.

**Results:**

Twenty cases of primary HBL (mean [SD] LDL-C: 42 [7] mg/dL) were diagnosed, leading to a prevalence of 2.39%. In comparison, the prevalence of HBL in a healthy control population was 0.57%. Psychiatric patients with HBL were characterized by a higher frequency of schizophrenia (*p*=0.044), hetero-aggression (*p*=0.015) and pervasive and specific developmental disorders (including autism) (*p*=0.011).

**Conclusions:**

The prevalence of HBL is 4-fold higher in psychiatric than in general population. More specifically, some statistically significant associations were found between low LDL-C concentrations and schizophrenia, autism and hetero-aggression. These data reinforce the hypothesis for a link between genetically low LDL-C levels and psychiatric disorders.

**Electronic supplementary material:**

The online version of this article (10.1186/s12944-018-0892-4) contains supplementary material, which is available to authorized users.

## Background

Hypobetalipoproteinemia (HBL) is defined by plasma concentrations of LDL-cholesterol (LDL-C) or apolipoprotein B (APOB) that are lower than the fifth percentile for age and sex [1, 2]. Many secondary causes, such as critical illness, lipid-lowering drugs or a strict vegan diet, can cause HBL. Familial hypobetalipoproteinemia (FHBL; OMIM#615558) is a co-dominant disorder whose frequency in the heterozygous form is estimated to be 1:1000-1:3000 [1, 2]. Heterozygotes for FHBL are often asymptomatic, with plasma LDL-C levels usually between 20 and 50 mg/dL [3]. FHBL has been associated with a longevity syndrome, mainly linked to cardiovascular protection. FHBL may be due to various mutations in genes affecting LDL-C metabolism: mainly *APOB* [1, 2], but also *PCSK9* [4, 5] or *ANGPTL3* [6]. In approximately 50% of FHBL cases, the genetic etiology remains to be determined [1, 2].

In a previous study aiming at identifying new genes in LDL-C metabolism, we recruited FHBL patients by screening the database of the Nantes University Hospital (HYPOCHOL study, NCT02354079) [7]. We were surprised to observe that ≈ 40% of patients were coming from the Psychiatry units, which were ranked first amongst the different clinical departments, even after excluding eating disorders. The literature was discordant regarding the putative relationship between low cholesterol levels and psychiatric disorders, such as violent behavior [8], suicide [9], impulsivity [10], autism [11] or mood disorders [12]. Based on this intriguing finding, we decided to conduct the HYPOPSY (HYPObetalipoproteinemia in PSYchiatric patients) study (NCT02889614). The objectives of the HYPOPSY study were to estimate the prevalence of primary HBL (defined by a plasma LDL-C concentration ≤ 50 mg/dL) in patients hospitalized in the psychiatric units of the Nantes University Hospital and to further investigate the related psychiatric disorders.

## Methods

### Participants

The HYPOPSY study was based on a retrospective medical and biological record review, and was approved by the local Research Ethics Committee. The retrospective and non-interventional design of this study made the consent of the patients unnecessary.

**Fig. 1:**
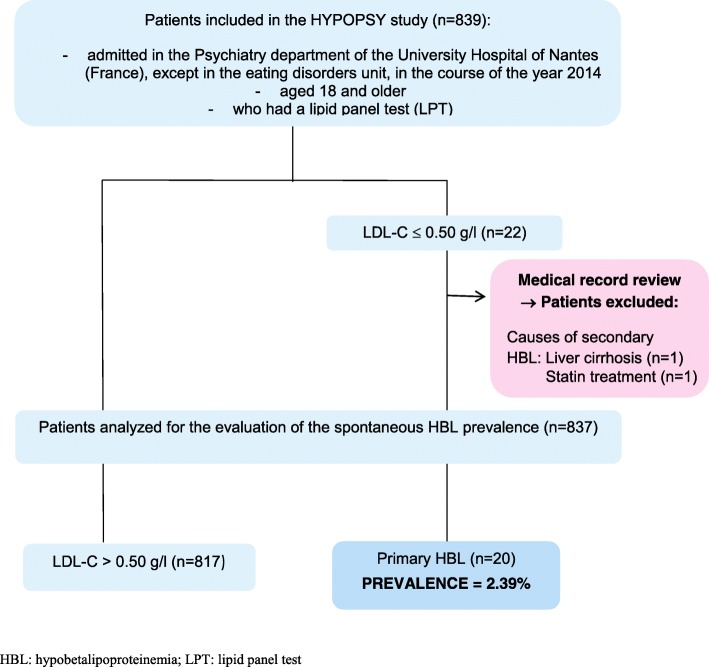
Flow chart of patients’ selection in the HYPOPSY Study

The flow chart of patients’ selection is presented in Fig. 1. To be included, participants had to be admitted in the Psychiatry department in the course of the year 2014, to be aged 18 and older, and to have had a lipid panel test (LPT). Patients admitted in the eating disorders unit were not included, because eating disorders are causes of starvation, leading to secondary HBL. Among the patients identified with a low plasma LDL-C concentration, other causes of secondary HBL were excluded by a thorough review of medical records: lipid-lowering drug treatment or low-fat diet, any cause of starvation, thalassemia or sickle-cell anemia, severe renal or pancreatic failure, decompensated liver failure and hyperthyroidism.

### Measures

For each patient included in the HYPOPSY study, a set of clinical and biological variables were systematically collected:

1. Demographic characteristics: age, sex.
2. Physical characteristics: weight, height, body mass index, current lipid-lowering drugs or low-fat diet, history of diabetes/other cardio-vascular risk factors or liver diseases.
3. Psychiatric characteristics: primary diagnosis and comorbid disorders according to the ICD-10 (International Classification of Disease – 10^th^ revision) [13], history of aggressive behaviors (hetero-aggression, suicidal attempts and other self-injuries), current psychotropic drugs use, familial history of psychiatric disorders.
4. Biochemical characteristics: LPT (measured plasma total cholesterol (TC), HDL-cholesterol (HDL-C) and triglycerides (TG) and calculated LDL-C according to the Friedewald formula), liver function test.

### Statistical analysis

#### Prevalence of primary HBL

A case of spontaneous HBL was defined as a plasma LDL-C concentration ≤50 mg/dL. The prevalence of primary HBL was assessed by the percentage of patients with HBL among whom having a LPT and without any medical cause of secondary HBL. The prevalence of HBL in the psychiatric population was compared to the one estimated in a general non-psychiatric population from the Health Care Center (Saint-Nazaire, France) of French Health Insurance between 2012 and 2014 (control population), in the framework of the HYPOCHOL study (with the same definition of HBL).

#### Psychiatric characterization of primary HBL

We considered two groups of patients (“Non-HBL” and “HBL”). An univariate analysis exploring links between the demographic, physical, psychiatric, and biological characteristics on the one hand, and the LDL-C status on the other hand, was performed using Mann-Whitney tests for the quantitative variables and Fisher tests for the qualitative variables.

The statistical analysis was carried out with SAS 9.1 and R statistical software (SAS Institute, Inc.).

## Results

### Prevalence of primary HBL in a psychiatric population

As shown in Fig. 1, 839 patients were included in the study, among whom 2 were excluded due to a medical cause of secondary HBL. The prevalence of primary HBL was estimated to 2.39 % in this psychiatric population, whereas the prevalence in the control population was estimated to 0.57% (Additional file 1: Table S1).

### Metabolic characteristics of patients with HBL

The anthropometric and metabolic characteristics of the patients with and without HBL are shown in Table 1. Patients with HBL were younger, with a lower body mass index, and had lower mean plasma TC, LDL-C and TG concentrations. Plasma lipids values were in the normal ranges in the “Non-HBL” group. There was no significant difference in the use of psychotropic drugs, including those known to interfere with lipid metabolism, between the two groups (Table 1).

**Table 1:** Comparison between HBL and non-HBL groups, according to demography and main biological features (*n*=837)

	HBL (*n*=20)	Non-HBL (*n*=817)	*p* - value^$^
Demography			
Age (± SD); years	35± 10	44± 14	0.0032^¶^
Gender (% Male)	65%	59%	0.36
Weight (± SD). kg	66 ± 11	72 ± 18	0.094
BMI (± SD). kg/m^2^	22 ± 4	25 ± 14	0.0115^¶^
Biological features (mg/dL)			
TC (± SD)	123 ± 28	186 ± 40	<0.0001^¶^
LDL-C (± SD)	42 ± 7	109 ± 34	<0.0001^¶^
HDL-C (± SD)	66 ± 30	53 ± 20	0.067
TG (± SD)	74 ± 52	115 ± 56	<0.0001^¶^
Psychotropic drugs			
Psychotropic drug consumption	45%	60%	0.17
Psychotropic drug with lipid metabolism impact^a^	25%	39%	0.25

^$^*p*-values obtained by Mann-Withney or Fisher exact tests; *HDL-C* high-density lipoprotein cholesterol; *LDL-C* low-density lipoprotein cholesterol, *TC* Total cholesterol, *TG* triglycerides

^a^these drugs include clozapine, olanzapine, risperidone, aripiprazole, zuclopenthixol, quetiapine, paliperidone, chlopromazine

^¶^*p*-value < 0.05

### Characteristics of psychiatric disorders associated to HBL

The distribution of psychiatric disorders in patients with and without HBL is shown in Table 2. Patients with HBL were characterized by a higher frequency of “pervasive and specific developmental disorders”, especially pervasive developmental disorders. Although there was no significant difference for the global category of “schizophrenia, schizotypal and delusional disorders”, a deeper analysis conducted on each detailed diagnosis indicated that patients with HBL were also characterized by a higher frequency of schizophrenia. The comparison of aggressive behaviors in patients with and without HBL is shown in Table 3. A higher proportion of hetero-aggression was found for patients with HBL.

**Table 2:** Comparison between HBL and non-HBL groups, according to the primary psychiatric (ICD-10) (*n*=837)

	HBL (*n*=20)	Non-HBL (*n*=817)	*p*-value
F00-09: Organic, including symptomatic, mental disorders	0 (0%)	10 (1%)	>0.99
F02: Dementia in other diseases	0 (0%)	2 (<1%)	>0.99
F03: Unspecified dementia	0 (0%)	5 (<1%)	>0.99
F06: Other mental disorders due to known physiological condition	0 (0%)	1 (<1%)	>0.99
F07: Personality and behavioral disorders due to known physiological condition	0 (0%)	1 (<1%)	>0.99
F09: Unspecified mental disorder due to known physiological condition	0 (0%)	1 (<1%)	>0.99
F10-19: Mental and behavioral disorders due to psychoactive substance use	4 (20%)	200 (24%)	0.79
F10: Alcohol related disorders	1 (5%)	126 (15%)	0.34
F11: Opioid related disorders	0 (0%)	3 (<1%)	>0.99
F12: Cannabis related disorders	1 (5%)	16 (2%)	0.33
F13: Sedative. hypnotic.or anxiolytic related disorders	0 (0%)	3 (<1%)	>0.99
F19: Other psychoactive substance related disorders	2 (10%)	53 (6%)	0.38
F20-29: Schizophrenia. schizotypal and delusional disorders	9 (45%)	291 (36%)	0.47
F20: Schizophrenia	8 (40%)	163 (20%)	0.044^¶^
F21: Schizotypal disorder	0 (0%)	5 (<1%)	>0.99
F22: Delusional disorders	0 (0%)	34 (4%)	>0.99
F23-24: Brief psychotic disorder / Shared psychotic disorder	0 (0%)	29 (4%)	>0.99
F25: Schizoaffective disorder. bipolar type	1 (5%)	41 (5%)	>0.99
F28: Other psychotic disorder not due to a substance or known physiological condition	0 (0%)	10 (1%)	>0.99
F29: Unspecified psychosis not due to a substance or known physiological condition	0 (0%)	9 (1%)	>0.99
F30-39: Mood [affective] disorders	3 (15%)	191 (23%)	0.59
F30: Manic episode	0 (0%)	17 (2%)	>0.99
F31: Bipolar disorder	3 (15%)	81 (10%)	0.44
F32: Major depressive disorder. single episode	0 (0%)	68 (8%)	0.39
F33: Major depressive disorder. recurrent	0 (0%)	20 (2%)	>0.99
F34: Persistent mood [affective] disorders	0 (0%)	2 (<1%)	>0.99
F38-39: Other mood [affective] disorders / Unspecified mood [affective] disorder	0 (0%)	4 (<1%)	>0.99
F40-48: Neurotic. stress-related and somatoform disorders	1 (5%)	83 (10%)	0.71
F40: Phobic anxiety disorders	0 (0%)	2 (<1%)	>0.99
F41: Other anxiety disorders	0 (0%)	21 (3%)	>0.99
F43: Reaction to severe stress. and adjustment disorders	1 (5%)	52 (6%)	>0.99
F44: Dissociative and conversion disorders	0 (0%)	5 (<1%)	>0.99
F48: Other nonpsychotic mental disorders	0 (0%)	3 (<1%)	>0.99
F60-69: Disorders of adult personality and behavior	1 (5%)	28 (3 %)	0.51
F60-61: Specific personality disorders/ mixed and other personality disorders	1 (5%)	26 (3%)	0.48
F66: Other sexual disorders	0 (0%)	1 (<1%)	>0.99
F69: Unspecified disorder of adult personality and behavior	0 (0%)	1 (<1%)	>0.99
F70-79: Mental retardation	0 (0%)	6 (<1%)	>0.99
F80-89: Pervasive and specific developmental disorders	2 (10%)	8 (<1%)	0.022^¶^
F83: Mixed specific developmental disorders	0 (0%)	1 (<1%)	>0.99
F84: Pervasive developmental disorders	2 (10%)	5 (<1%)	0.011^¶^
F88: Other disorders of psychological development	0 (0%)	2 (<1%)	>0.99

^¶^*p*-value < 0.05

**Table 3:** Comparison between HBL and non-HBL groups, according to the history of aggressive behaviors (*n*=837)

	HBL (*n*=20)	Non-HBL (*n*=817)	*p*-value^*^
Hetero-aggression	5 (25%)	60 (7%)	0.015^¶^
Suicidal attempts (current or lifetime)	1 (5%)	60 (7%)	>0.99
– Violent suicidal attempts	0 (0%)	17 (2%)	>0.99
– Non-violent suicidal attempts	1 (5%)	45 (6%)	>0.99
Other self-injuries	0 (0%)	3 (<1%)	>0.99

*p-values obtained with a Fischer exact test

^¶^*p*-value < 0.05

## Discussion

We found a 4-fold higher prevalence of HBL in a psychiatric population than in a control population (2.39 vs 0.57%). HBL patients were characterized by a higher prevalence of schizophrenia and hetero-aggressive behaviors compared to control patients. In addition, while the absolute number was low (*n*=2), there was also a higher prevalence of pervasive developmental disorders in patients with HBL.

To the best of our knowledge, this is the first time that the prevalence of HBL was specifically assessed in such a broad psychiatric population. In order to identify HBL patients, we used a LDL-C concentration ≤ 50 mg/dL as a cutoff, instead of an adjusted value of LDL-C ≤ 5^th^ percentile for age and sex [1, 2]. We used this cutoff since FHBL patients usually have LDL-C values between 20 and 50 mg/dL [3, 14]. With these diagnosis criteria, the prevalence of HBL in the control population was 0.57%, which is moderately higher than the classically reported frequency of FHBL (1/1000 to 1/3000) [14, 15]. One striking finding was the very high frequency of HBL in patients hospitalized in Psychiatry department, with more than one patient of 50 who exhibit spontaneous low LDL-C levels. This result is in line with the report of Huang *et al.* that found that serum TC levels in 213 psychiatric inpatients were significantly lower than control values [16].

Importantly, we carefully excluded patients under lipid-lowering drugs (mainly statins) as well as secondary causes of HBL. As it could be a potential cause of false positive results, we also verified that there was no imbalance between the use of antipsychotic drugs (with a specific focus on those interfering with lipid metabolism: clozapine, olanzapine, risperidone, aripiprazole, zuclopenthixol, quetiapine, paliperidone, chlopromazine) between patients with and without HBL. Moreover, antipsychotic medications, especially second-generation antipsychotics, are often associated with increased TG and, in a lesser extent, increased LDL-C levels but not with low LDL-C concentrations [17].

We also determined that pervasive developmental disorders, schizophrenia and hetero-aggressive behaviors were more frequent among psychiatric patients with HBL. These data reinforce the hypothesis for a link between genetically low LDL-C levels and violent behavior or impulsivity.

In the literature, several studies have highlighted a potential link between low cholesterol levels and violence, including hetero-aggression and suicides [18]. The evidence for such association mostly derives from epidemiological and observational studies in general and violent populations. For instance, a meta-analysis of 18 epidemiological studies found 50% more violent deaths in men with TC levels less than 160 mg/dL than in subjects with higher TC levels [19]. A prospective study conducted in 6393 working men followed for up to 17 years also found a 3.16 (95% CI: 1.38 to 7.22, *P* = 0.007) increase in relative risk of death from suicide in subjects with low average serum TC concentration (< 185 mg/dL) compared with those with average serum TC concentration of 185 to 240 mg/dL [20]. In addition, a study has also showed that low TC levels were associated with increased impulsivity in 301 patients referred to a psychiatric clinic [10]. Old meta-analyses of randomized trials with cholesterol-lowering drugs tended to identify a trend for more violent deaths in treated *vs* control groups [18, 21, 22]. However, these initial findings were not further supported in more recent trials with statins, even in those with the highest doses and thus the lowest concentrations of LDL-C [23]. Very recently, no psychiatric safety signal was observed in the FOURIER cardiovascular outcomes trial with evolocumab, a PCSK9 monoclonal antibody, in which patients in evolocumab group achieved the lowest LDL-C concentration (*i.e.* 30 mg/dL) never reached in previous clinical studies [24, 25].

The literature is scarce regarding the link between FHBL and impulsive and/or violent behaviors. However, a case report of a patient with a heterozygous mutation of *APOB* (apoB29.4) causative of FHBL highlighted a potential association between the hypocholesterolemic status of family members and violent behavior, with an odds ratio of 16.9 [26]. In the HYPOPSY study, genetic analysis was not performed in patients with HBL, thus we were unable to perform some genotype-phenotype correlations. We plan to conduct a prospective study in the same psychiatric population in which genetic analyses and family inquiries will be performed in patients with HBL in order to determine whether specific mutations in lipoprotein metabolism predispose to psychiatric disorders.

Finally, some experimental studies have demonstrated an increase in violent behavior in monkeys assigned to low-cholesterol diet [27, 28]. The suspected underlying molecular mechanism is the reduction of brain serotonin transmission that has been demonstrated to be associated with increased impulsivity [29, 30]. Several studies have suggested that plasma cholesterol could be a marker for central serotoninergic activity [31, 32] and monkeys fed with a low-cholesterol diet had lower concentrations of serotonin metabolites (5-HIAA) in their cerebrospinal fluid than monkeys on high-cholesterol diet [28].

In contrast to violent behaviors, there was no previously published report for a specific association between FHBL and schizophrenia. To the best of our knowledge, only the study of Atmaca *et al.* demonstrated that serum TC levels were lower in medication-free schizophrenic patients compared to healthy controls [33], even though the number of patients was very low (*n*=16 for each group). However, we cannot exclude a reverse causality between schizophrenia and low LDL-C levels since a Japanese study has found that the prevalence of hypoproteinemia and hypocholesterolemia were significantly higher in schizophrenia patients than in age- and sex-matched healthy volunteers [34]. Thereby, a precise nutritional status assessment will be required in the future prospective study to exclude that HBL could be linked to undernutrition in psychiatric population.

Our result about the potential link between the significant association between HBL and pervasive developmental disorders has to be interpreted cautiously due the very low number of cases. Autism spectrum disorders and schizophrenia are two complex psychiatric conditions with significant heritability, and which share common phenotypic (*i.e.* aggressive behaviors) and endophenotypic (*i.e.* neurocognitive anomalies such as alterations in executive functions and social functioning) characteristics. Interestingly, a study found that 19% of children with family history of autism spectrum disorders had low TC levels (*i.e.* < 100 mg/dL) [11]. Moreover, a very recent study conducted in 23 male with Fragile-X syndrome, one of the main genetic cause of autism and intellectual deficiency, showed that these subjects had statistically significant lower levels of all lipid parameters as compared to control individuals [35]. Some additional dedicated studies performed in this specific population are warranted to confirm or invalidate the association between low LDL-C levels and autism spectrum disorders.

The results of our study should be interpreted in the light of several methodological limitations, which may also serve as lessons for the preparation of future works in the field. The retrospective design of the study can have introduced some bias, especially regarding the etiology of HBL. Notably, it cannot be excluded that some secondary causes of low LDL-C levels were missed (hyperthyroidism, undernutrition, etc.). In the same way, we did not have any data regarding the familial history and genetic analysis of HBL. Finally, this is a cross-sectional study and we collected only one LDL-C value to assess the prevalence of HBL. It should be interesting to have several LDL-C values, as well as APOB levels, in order to confirm FHBL diagnosis.

In contrast, the strengths of this study are: i) the large size of our “real-life” cohort (> 800 patients); ii) the use of a comparative control cohort in general population; iii) the precise identification of psychiatric disorders using the ICD-10 classification.

However, additional prospective studies need to be conducted to confirm the higher prevalence of FHBL in the psychiatric population and to perform more extensive genotype-phenotype comparisons. Such studies are desirable regarding the safety of extremely low LDL-C levels achieved with the new classes of lipid-lowering drugs [36].

## Conclusions

The HYPOPSY study found a ≈4 fold higher prevalence of HBL in patients hospitalized in Psychiatry department than in a general healthy population. More specifically, some statistically significant associations were found between low LDL-C concentrations and schizophrenia, autism and hetero-aggression. These data reinforce the hypothesis for a link between genetically low LDL-C levels and violent behavior or impulsivity. However, additional prospective studies need to be conducted in this Psychiatric population to confirm the higher prevalence of FHBL and to perform more extensive genotype-phenotype comparisons. Such studies are desirable regarding the safety of extremely low LDL-C levels achieved with the new classes of lipid-lowering drugs [36]. The findings of the HYPOPSY study has served to establish power calculation, to evaluate methodological issues and to better specify which data to be collected for the preparation of a larger prospective research program named PARTITION (Prevalence of fAmilial hypobetalipopRoTeinemIa in psychiaTrIc pOpulatioN) and conducted in the framework of a larger project: the CHOPIN (CHOlesterol Personnalized Innovation) program. The PARTITION study aims at better characterizing the links between HBL and psychiatric disorders, and at evaluating a possible genotype-phenotype relationship between identified mutations and the severity of psychiatric symptoms.

## Additional file

Additional file 1: **Table S1.** Prevalence of HBL patients in the control population (Health care center of St Nazaire) (DOCX 22 kb)

## Abbreviations

APOB: Apolipoprotein B
CHOPIN: CHOlesterol Personnalized Innovation
FHBL: Familial hypobetalipoproteinemia
HBL: Hypobetalipoproteinemia
HDL-C: HDL-Cholesterol
HYPOPSY: HYPObetalipoproteinemia in PSYchiatric patients
ICD-10: International Classification of Disease – 10^th^ revision
LDL-C: LDL-Cholesterol
LPT: Lipid Panel Test
PARTITION: Prevalence of fAmilial hypobetalipopRoTeinemIa in psychiaTrIc pOpulatioN
TC: Total Cholesterol
TG: Triglyceride
