# Supplementary material for: Prevalence of hypobetalipoproteinemia and related psychiatric characteristics in a psychiatric population: results from the retrospective HYPOPSY Study
Source: Lipids Health Dis. 2018 Nov 6;17:249. doi: 10.1186/s12944-018-0892-4 (PMC6220563; doi:10.1186/s12944-018-0892-4)
Supplement: Supplementary file 1 — Table S1. Prevalence of HBL patients in the control population (Health care center of St Nazaire) (DOCX 22 kb) [file 12944_2018_892_MOESM1_ESM.docx]

**Supplemental Table 1. Prevalence of HBL patients in the control population (Health care center of St Nazaire)**

|  | **HBL** | **Non-HBL** |
| --- | --- | --- |
| ***Year*** |  |  |
| 2012 (number of patients, %) | 44 (0,70%) | 6265 (99,30%) |
| 2013 (number of patients, %) | 41 (0,61%) | 6767 (99,39%) |
| 2014 (number of patients, %) | 26 (0,40%) | 6455 (99,60%) |
| ***Average over 3 years*** | 111 (0,57%) | 19487 (99,43%) |
